# Vanadium’s journey from essential trace metal to emerging environmental pollutant in the Anthropocene era

**DOI:** 10.55730/1300-0527.3785

**Published:** 2026-02-10

**Authors:** Tanu ARORA, Nivedita AGNIHOTRI, Mohammad AZAM

**Affiliations:** 1 Department of Chemistry, Maharishi Markandeshwar (Deemed to be University), Mullana, Ambala, India; 2 Department of Chemistry, College of Sciences, King Saud University, Riyadh, Saudi Arabia

**Keywords:** Vanadium, speciation, toxicity, analytical methods, redox chemistry, circular economy

## Abstract

Vanadium, previously considered a minor trace element, is gaining global environmental attention due to its expanding applications in metallurgy, energy storage, catalysis, and agriculture. With this rise in its industrial and technological uses, however, anthropogenic emissions of vanadium into the atmosphere, soils, and water bodies have also escalated. Unlike many heavy metals, vanadium exhibits complex redox behavior, primarily existing as V(IV) and V(V) species, which significantly impacts its environmental mobility, bioavailability, and toxicity. Vanadium exhibits complex behavior that necessitates an integrated research approach to better understand its various roles in scientific fields. This review summarizes the current knowledge on vanadium’s environmental presence, transport, chemical forms, and ecological impacts, investigating primary sources like fossil fuel combustion and mining, as well as innovative technologies such as vanadium redox flow batteries. The study highlights vanadium’s toxic effects on aquatic life, plants, and soil microbes, addressing risks linked to different chemical forms and chronic exposure gaps. It also discusses human exposure and regulatory disparities alongside challenges in analytical methods for measuring trace vanadium fluxes. In this review, “vanadium speciation” denotes the occurrence of vanadium across different oxidation states, coordination environments, and environmental phases. The study concludes by highlighting important research gaps in the areas of long-term ecological effects, microbiological interactions with vanadium, and the environmental consequences of recovering and recycling vanadium within the context of a circular economy. This places vanadium in the Anthropocene era as a trace metal that is evolving from an industrial element to an emergent environmental pollutant.

## 1. Introduction

Vanadium is a transition metal commonly found throughout Earth’s crust, usually present in soils and sediments at trace levels of 100 to 200 mg kg^−1^ [1,2]. It naturally occurs in minerals such as vanadinite and carnotite and is also present in small amounts in fossil fuels, including crude oil and coal [3]. Traditionally, vanadium has been considered of limited environmental concern due to its generally low concentrations and minimal bioavailability in natural settings. However, its unique chemical properties, especially its ability to exist in multiple oxidation states, are attracting growing scientific interest [4].

In recent years, the industrial use of vanadium has increased significantly due to its important roles in the production of steel alloys, catalysts, pigments, and fertilizers [5] and especially in new energy storage systems like vanadium redox flow batteries (VRFBs) [6]. The increasing adoption of VRFBs, valued for their long lifespan and scalability in grid energy storage, has led to a rise in global vanadium demand and heightened concerns about environmental emissions associated with their extraction, processing, and disposal [7].

Human activities release vanadium primarily through the combustion of fossil fuels, in addition to mining and metallurgical processes [8,9]. Vanadium exists in two main common oxidation states when released into the environment: vanadate and vanadyl. These forms exhibit variations in mobility, toxicological risk, and biological accessibility. Complex redox mechanisms, driven by determinants such as organic matter, pH, and microbial interactions, significantly affect the environmental dynamics [10].

Vanadium’s impact on the environment and human health is worsening as human use of the metal continues to rise. Studies have shown that vanadium is harmful to humans, plants, aquatic life, and soil microbes. Its effects include disrupting the essential metal balance, causing oxidative stress, and inducing cell toxicity [11,12]. Despite these dangers, vanadium remains an underrecognized contaminant in many regulatory systems, with insufficient environmental monitoring and inconsistent global guideline levels [13].

This review aims to comprehensively compile current knowledge on vanadium’s environmental sources, transportation and dispersion, speciation dynamics, redox chemistry, and toxicological and ecotoxicological effects; advanced analytical techniques for vanadium speciation and detection; and regulatory standards and environmental guidelines. Furthermore, it aims to identify significant research gaps and suggest future directions to improve the understanding and management of vanadium as a rising environmental contaminant in the Anthropocene era [14].

## 2. Origin of vanadium in nature

Vanadium is introduced into the environment through multiple pathways arising from both inherent geological processes (e.g., weathering of vanadium-bearing rocks, volcanic activity, sedimentary processes, hydrothermal activity, tectonic activity, or metamorphism) [15] and anthropogenic activities (e.g., coal combustion, oil combustion, industrial processes, or waste disposal) [16,17]. A thorough understanding of the importance of these sources is essential for accurately assessing the environmental distribution of vanadium, pinpointing areas with higher contamination, and evaluating the related ecological and human health risks [9,18,19]. Although natural sources guarantee a baseline presence of vanadium, anthropogenic emissions have progressively modified its environmental concentrations and spatial distribution, thereby intensifying concerns regarding its environmental behavior and impact across various ecosystems [19,20].

### 2.1. Natural sources

Vanadium is not inherently found in its elemental form in nature but is widely distributed as one of the minor constituents of Earth’s crust. At high concentrations, it occurs in ores, numerous minerals, natural materials, and certain specialized deposits [21,22]. Nevertheless, vanadium is ubiquitously present in the natural environment, primarily introduced through the weathering and erosion of vanadium-bearing minerals. Table 1 provides an overview of selected vanadium-bearing minerals, outlining their chemical formulas, typical geological settings, and supporting references [23–26].

**Figure 1:**
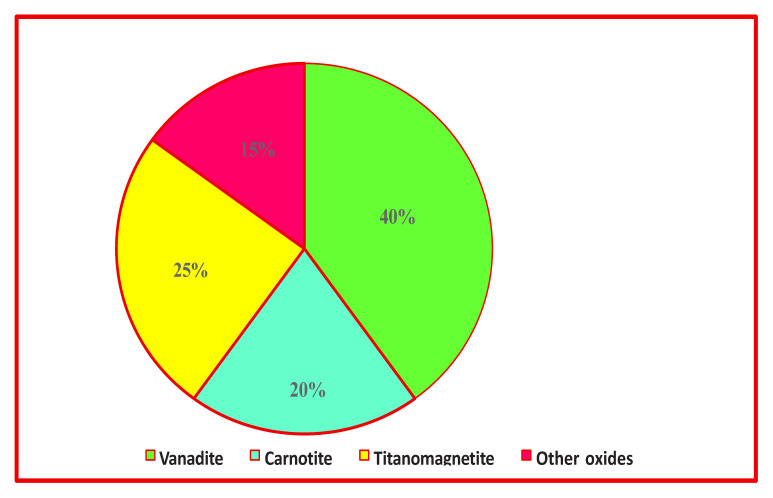
Natural occurrence and relative abundance of vanadium-bearing minerals.

These minerals release vanadium ions into nearby soils and water bodies over long geological periods through gradual dissolution processes [27]. Additionally, certain geological materials, including black shales and specific igneous and sedimentary rocks, serve as important reservoirs of vanadium, influencing the vanadium levels of regional soils and sediments [28,29]. Moreover, vanadium is naturally present in soils and sediments worldwide, although its concentrations vary considerably depending on local geological, mineralogical, and soil characteristics [30]. Figure 1 and Table 1 illustrate the geological distribution and host mineral phases of vanadium, providing insight into its occurrence patterns relevant to exploration and resource assessment.

Volcanic activity is another significant natural source, with eruptions releasing vanadium-rich particulates and gaseous compounds into the atmosphere [31]. The transboundary dispersion of these emissions allows vanadium to be widely deposited, affecting terrestrial and aquatic environments far from the volcano [32,33]. This naturally occurring baseline level of vanadium provides a reference point for evaluating human-made contributions [34,35].

### 2.2. Anthropogenic sources

Human activities have significantly increased environmental vanadium levels beyond natural background levels. The primary human-related source is the burning of fossil fuels, such as heavy oils, coal, and crude oil, which naturally contain high vanadium levels due to their geological origins [36]. Combustion in power plants, industrial boilers, and vehicle engines releases vanadium-enriched aerosols and particulates into the atmosphere. These emissions are then deposited onto soils and into water systems, increasing vanadium buildup at local and regional levels [8,30].

**Figure 2:**
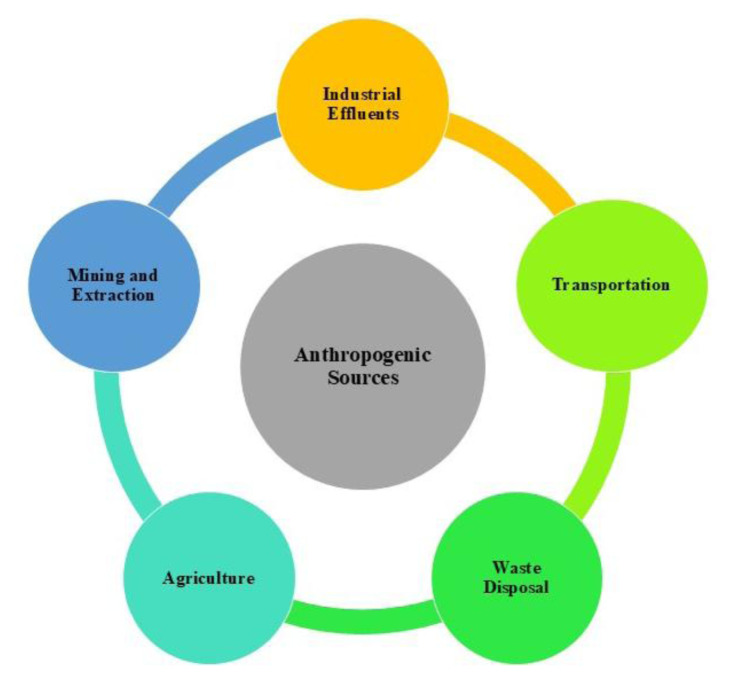
Anthropogenic sources of vanadium in the environment.

The mining and metallurgical sectors also significantly contribute to vanadium pollution, especially in regions where vanadium ores are extracted and processed [37]. During ore beneficiation and smelting, vanadium can be released as dust or discharged in effluents, increasing the risk of environmental contamination in nearby areas. Additionally, vanadium’s widespread use as an alloying element in steel production, intended to enhance strength and corrosion resistance, results in industrial wastes containing vanadium, which can enter the environment if not properly controlled [30]. Figure 2 illustrates the range of human activities that increase the presence of vanadium in the environment.

Recent advancements in vanadium-based technologies, especially VRFBs used for large-scale energy storage, are emerging as potential sources of environmental vanadium pollution [38]. Without proper containment, the production, operation, and disposal of VRFBs may lead to localized vanadium emissions. Additionally, the use of vanadium-enriched fertilizers and the processing of electronic wastes are recognized as further human activities capable of increasing vanadium levels in soils and aquatic systems [39,40]. These recently emerging sources of contamination are increasingly recognized for their role in enhancing vanadium’s mobility and bioavailability within ecosystems, highlighting the urgent need for robust monitoring and control measures.

### 2.3. Vanadium’s environmental transformation and dispersion

The fate of vanadium in the environment is mainly determined by its speciation, redox transformations, and interactions with both living and nonliving environmental components, which collectively affect its environmental behavior and distribution in different media [41,42]. Depending on the physicochemical environment, vanadium adopts various oxidation states, which promote its transformation and partitioning across different aqueous, solid, and gaseous media [43]. These transformations determine how vanadium disperses through different pathways, including soils, sediments, and aquatic bodies, ultimately affecting its bioavailability and ecological impact [10].

#### 2.3.1. Environmental transformation of vanadium

In the environment, vanadium undergoes complex biogeochemical processes primarily controlled by redox conditions, pH levels, and microbial activity. Its speciation exhibits temporal changes among V(III), V(IV), and V(V), each characterized by its own unique environmental behavior and toxicological profile [44,45].

In aerobic environments, pentavalent vanadate ions, primarily monohydrogen vanadate ions and dihydrogen vanadate ions, exhibit high miscibility, enabling significant mobility in water bodies. Despite their tendency to adhere to mineral surfaces, these forms remain bioavailable and toxic, posing risks to ecosystems and human health [46]. Conversely, in anaerobic settings such as submerged soils and sediments, vanadium exists in the lower oxidation states of V(III) and V(IV). These reduced forms are more likely to bind with sulfide phases, metal oxides, and organic substrates, effectively sequestering vanadium and reducing its environmental availability [47,48].

Microorganisms have a remarkable influence on vanadium-based redox processes, with some bacteria reducing pentavalent vanadium to V(IV) or V(III) as an outcome of their metabolic pathways. This contributes to vanadium sequestration in oxygen-depleted environments, helping to regulate vanadium’s mobility and retention in anoxic settings. Under oxygen-rich conditions, oxidative microbes convert reduced vanadium forms back to the pentavalent state, modulating its mobility, bioavailability, and ecological consequences. Aerobic microbial communities reoxidize vanadium, supporting redox cycling and influencing its speciation, distribution, and toxicity in soil and water systems [49]. This biological transformation pathway controls vanadium’s environmental behavior and toxicological effects.

By altering the prevalent chemical forms and their sorption affinities, pH significantly influences the speciation of vanadium. Compared to anionic vanadate, which is often found at neutral to alkaline pH levels, vanadium tends to form neutral or cationic species with lower mobility in acidic conditions [50]. Understanding these fundamental biological and physicochemical processes is crucial for predicting vanadium’s environmental behavior and developing effective remediation strategies.

#### 2.3.2. Environmental dispersion of vanadium

##### 2.3.2.1. Vanadium fluxes derived from the lithosphere

Weathering processes are essential for the natural transfer of vanadium from lithosphere sources. Physical erosion mobilizes approximately 1.63 × 10^12^ g/year, mainly as particulate matter transported by rivers. In contrast, chemical dissolution releases about 2.1 × 10^10^ g annually based on steady-state V/Si ratios observed in global river systems. Volcanic outgassing and wind-driven dust transport are smaller but measurable sources of natural vanadium fluxes [51].

##### 2.3.2.2. Influence of human activities on vanadium release

Anthropogenic inputs now dominate the global vanadium budget. Each year, approximately 7.3 × 10^11^ g of vanadium are introduced into the environment through industrial processes. Mining (18%), petroleum processing (56%), and coal combustion (26%) are the principal contributors. The sharp increase in fossil fuel usage, particularly vanadium-rich crude oils, has led to heightened atmospheric deposition and environmental dispersion of vanadium [51].

Human activities have greatly intensified the movement and spread of vanadium across environmental domains, often surpassing natural background dynamic exchanges. Significant sources include the burning of fossil fuels, mining and metallurgical operations, industrial effluents, and waste disposal. The principal contributors to atmospheric vanadium pollution are the burning of vanadium-laden fossil fuels, particularly heavy crude oils and bituminous coals. Vanadium pentoxide, a particle that shows mobility at long distances before it settles on land and water, is the primary type of emission of volatilized vanadium compounds during combustion. The increased reliance on unconventional petroleum sources, including tar sands and petroleum coke, has further worsened global vanadium emissions, significantly increasing atmospheric deposition [51,52]. The mining and smelting of vanadium-rich ores generate vanadium-containing pollutants, including dust, slag, and effluents, that can contaminate soil and groundwater, as well as causing problems with wastewater management. This pollution is exemplified by the production of ferrovanadium alloys from titanomagnetite ores in industrial sectors [46].

Beyond major industrial sources, vanadium is released into the environment in smaller amounts through urban and agricultural sources. Emissions from vehicles and industries, along with the breakdown of construction materials and agrochemicals, contribute to minor runoff, collectively adding to environmental inputs [53]. Notably, vanadium mainly exists in its highly soluble and toxic pentavalent oxidation state, which results in greater mobility and bioavailability compared to its naturally occurring reduced forms. This increases the risk to ecology and human health from vanadium exposure. Therefore, understanding these human-made pathways and vanadium’s environmental behavior is essential for conducting risk assessment and developing appropriate mitigation strategies [49].

## 3. Speciation dynamics and redox chemistry of vanadium

In order to fully comprehend the biochemical effects of elements on life, it is essential to correctly quantify the species that are formed within a certain environmental or biological matrix. Speciation is the process of identifying and measuring one or more different chemical species in a sample. Despite being abundantly spread across Earth’s crust, vanadium is frequently found in only trace amounts. The unique property of vanadium to exist in multiple oxidation states, ranging from +2 to +5, is a fundamental characteristic that influences the environmental chemistry of this metal. Because of their relative thermodynamic stability, vanadium predominantly exists in its higher oxidation states, specifically +4 and +5, under typical environmental conditions [54,55]. This characteristic of vanadium plays an essential role in shaping its redox dynamics and affects its environmental transport, biological uptake, and toxicological impact in aqueous environments. In oxidation state +5, vanadium inhibits phosphorylases such as protein tyrosine phosphatase and ATPase. It may be necessary for humans despite being poisonous at higher concentrations as it is a cofactor for various enzymes, such as nitrogenases and haloperoxidases. Vanadium can interact with membrane interfaces and break DNA, changing the characteristics of its coordination complexes. Research has demonstrated the behavior of vanadium coordination complexes in biological fluids, emphasizing the importance of speciation in shaping the impacts of metal compounds in biological systems and underscoring the need for more knowledge on biometal compartmentalization and vanadium localization. Thorough speciation profiling is thus essential for assessing the metal’s environmental fate and determining the risk levels [11,16]. Since variations in oxidation state, coordination environment, and solubility control cellular absorption, redox cycling, and biomolecular interactions, vanadium toxicity is intrinsically speciation-dependent. Therefore, a crucial framework for evaluating toxicological results is provided by comprehending vanadium speciation under environmentally relevant conditions.

### 3.1. Hydrolysis in aqueous chemistry

Of the several oxidation states of vanadium, biological systems are primarily found to have tetravalent and pentavalent states. These two states show complex chemistry in aqueous solutions, creating distinct species depending on their hydrogen ion concentration. Dimerization causes restricted VO^+^ cations to form at neutral pH levels, whereas diverse vanadate oligomers form at lower pH levels. Decavanadate can interconvert with oligomeric vanadates and is a yellow-orange substance that occurs in acidic environments. Alternatively, vanadium mainly exists as a tetravalent vanadyl ion under anoxic conditions, forming more stable complexes but reducing its environmental dispersion. Different species can be seen using methods like ^51^V NMR spectroscopy and electron paramagnetic resonance.

### 3.2. Redox behavior

Vanadium’s redox chemistry is extremely complicated and dependent on a number of variables such as hydrogen ion concentration, presence of chelating agents or oxygen, and metal concentration. V(IV) solutions are stable in acidic environments and found to be resistant to oxidation, but as the pH rises above 4, these solutions oxidize to V(V), generating VO(OH)^3−^ species at pH levels above 10. V(V) can be converted to V(IV) in reducing conditions, although it stays stable over a wide pH range. Glutathione, cysteine, and ascorbate are examples of biological reductants. Research indicates that V(IV) forms a variety of complexes, although there is conflicting information regarding V(V) reduction with glutathione, which depends on pH and concentration. Furthermore, vanadium’s speciation affects enzyme inhibition in phosphorylation processes, which is important for biological responses. More research is needed to fully comprehend its intricate interactions in biological fluids. These redox-driven transformations govern the distribution of vanadium among soluble and particulate forms, acting as a vital factor in controlling its ecological movement and bioaccumulation potential [56,24].

### 3.3. Molecular complexation and ligand dynamics

Complexation of vanadium is frequently observed with various ligands, including both organic and inorganic types, such as flavonols, humic acids, phosphates, and sulfates, which significantly alter its distribution and mobility [57–61]. This can enhance the solubility of vanadium and further increase its mobility, while competition for adsorption with other oxyanions influences its bioavailability and toxic effects [55]. Transient speciation equilibria are demonstrated within complex relationships involving hydrogen ion concentration, oxic–anoxic conditions, concentrations of complexing agents, and microbial activity, which complicate efforts in ecological modeling and vulnerability assessments [62].

## 4. Vanadium exposure: toxicological effects and ecotoxicology

Several factors, including speciation, amount, and length of exposure, influence the environmental and biological effects of vanadium. At higher concentrations, the pentavalent form of vanadium can have a significant ecotoxicological impact on multiple ecological levels, although it functions as a micronutrient for certain taxa.

### 4.1. Aquatic toxicity

According to Gustafsson [41], vanadium is particularly harmful to aquatic life, and its effects vary depending on the species, developmental stage, and water chemistry. Since vanadate ions structurally resemble phosphate, they pose a serious risk because they can replace phosphate in essential metabolic pathways. Oxidative stress, cellular metabolism, and enzymatic processes can all be affected by this interference [63,64]. Experimental studies have shown that exposure to vanadium causes significant changes in hematological parameters, disruptions in ionic regulation, and morphological damage to fish gill tissues [65,66]. Additionally, aquatic invertebrates and photosynthetic microorganisms are vulnerable to vanadium poisoning [67]. In freshwater algae, prolonged exposure to vanadium has been shown to inhibit growth and reduce photosynthetic efficiency. The accumulation of vanadium in the soft tissues of aquatic invertebrates, such as bivalves and crustaceans, also raises concerns about long-term ecological consequences and the potential for trophic transmission [68,69].

### 4.2. Environmental stress on soil and flora

According to Hanus-Fajerska et al. [11], vanadium causes phytotoxicity in terrestrial ecosystems by disrupting essential physiological processes such as photosynthesis, root growth, and seed germination. Several soil parameters, including pH, organic matter content, and the presence of competing anions like phosphate and sulfate, influence vanadium bioavailability and absorption, thereby affecting its toxicity to plants [39]. The ability of certain plant species to transport and accumulate vanadium in aboveground tissues raises concerns about its long-term effects on soil health and food security, as well as its entry into the food chain [70]. Elevated vanadium levels can also affect soil microbial communities; long-term exposure has been associated with changes in community composition, reductions in microbial biomass, and suppression of enzyme activities. According to Xiao et al. [71], these changes indicate potential damage to essential soil processes and overall ecosystem health.

### 4.3. Impact on human and animal health

Vanadium exposure has been associated with a variety of harmful health effects in human and animal populations, including organ damage, metabolic disturbances, and disruptions in essential functions [72].

#### 4.3.1. Systemic toxicity of vanadium in humans: a multiorgan perspective

Vanadium, a silvery-gray transition element extensively used in industrial processes, particularly catalytic oxidation, petrochemical refining, and alloy production, has emerged as a key environmental and occupational health concern. Vanadium pentoxide is considered the most dangerous among its various chemical forms, especially when inhaled. Workers in steel production, fossil fuel burning, and catalyst processes are more likely to be exposed to higher risks [73].

##### 4.3.1.1. Respiratory system involvement

The inhalation route serves as the primary pathway for vanadium entry in occupational environments. Numerous studies have documented its adverse effects on the respiratory tract, ranging from mild mucosal irritation to severe lower airway inflammation. The reported clinical symptoms include nasal inflammation (rhinitis), throat irritation (pharyngitis), dry cough, and bronchial discomfort. In cases of prolonged or high-level exposure, inflammatory processes can develop into more severe conditions such as tracheobronchitis or bronchopneumonia [74]. These respiratory insults are mainly caused by oxidative injury and immune activation triggered by reactive oxygen species produced during vanadium metabolism, which disrupt epithelial barriers and lead to leukocyte infiltration.

##### 4.3.1.2. Hematological and immune disruption

Vanadium adversely affects hematological stability by causing oxidative damage to red blood cell membranes, ultimately leading to hemolysis and a reduced lifespan of cells. Clinical signs include decreased erythrocyte counts, lower hematocrit, and decreased hemoglobin [75]. Additionally, vanadium impairs iron utilization and interferes with the regulatory role of erythropoietin, which worsens anemia. Its immunotoxic effects are reflected in changes in leukocyte subpopulations and reduced immune function, observed in both humans and laboratory models [76].

##### 4.3.1.3. Neurotoxicity and behavioral impairment

Substantial experimental evidence shows that vanadium harms the central nervous system. Behavioral tests conducted with rodents revealed memory problems, learning difficulties, and decreased activity after subchronic exposure [77]. Mechanistically, vanadium can cross the blood–brain barrier, triggering neuroinflammatory responses, interfering with mitochondrial energy production, and damaging neuronal membranes through peroxidation. These neuropathological effects are similar to those seen in neurodegenerative diseases like Alzheimer and Parkinson disease, suggesting that vanadium may also play a role in their development [78].

##### 4.3.1.4. Reproductive and embryotoxic outcomes

The reproductive and developmental systems are especially susceptible to damage caused by vanadium. In vivo investigations have linked maternal vanadium exposure during pregnancy to impaired fetal development, including reduced fetal weight, skeletal anomalies, and, in cases of severe exposure, fetal mortality [79]. Vanadium also harms male reproductive health by causing testicular atrophy, reduced sperm production, and problems with spermatogenic processes. These effects are connected to oxidative stress, hormonal disruption, and direct toxic effects on germ cells [80]. Given the rising environmental levels of vanadium, these findings emphasize its risk to reproductive fitness, especially in populations more heavily exposed to it.

##### 4.3.1.5. Renal and hepatic compromise

Both the liver and kidneys are primary sites for vanadium accumulation and are important targets of its systemic toxicity. Renal pathology includes glomerular inflammation, tubular necrosis, and decreased filtration ability [81]. Meanwhile, hepatic effects include cellular swelling, inflammatory infiltration, and increased activity of liver enzymes such as alanine transaminase and aspartate transaminase, which indicate hepatocellular stress [82]. Chronic vanadium accumulation in these organs raises the risk of cumulative damage, making long-term exposure particularly dangerous. Thus, vanadium shows a wide range of toxicological effects, impacting the respiratory, hematological, neurological, reproductive, renal, and hepatic systems. The primary mechanisms are mainly oxidative, emphasizing its role as a redox-active toxicant that can disturb cellular balance [83]. Although vanadium’s presence in the environment is increasing, it does not receive the same regulatory scrutiny as metals like arsenic or lead [84]. Future efforts should focus on understanding its mechanisms of action, establishing strict exposure limits, and improving surveillance in high-risk occupational settings to protect public health.

#### 4.3.2. Toxicological impacts of vanadium in animal models

**Figure 3:**
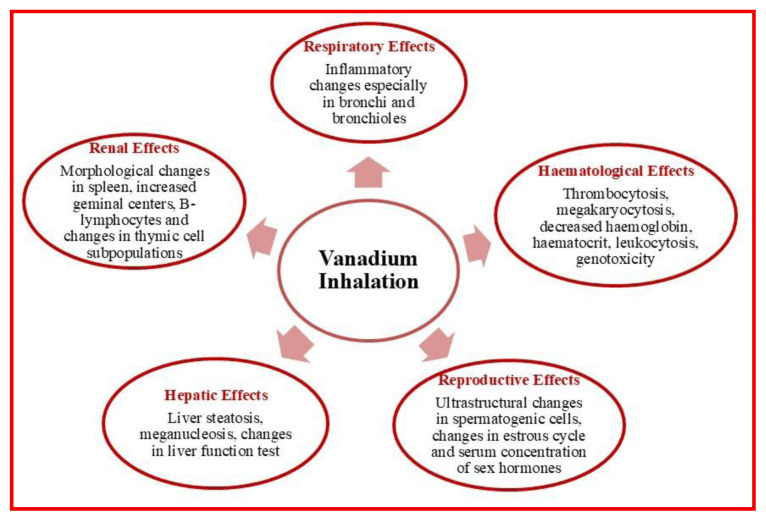
Effects of vanadium toxicity in human and animal models.

Vanadium has species-specific toxic effects in animals, with severity and health outcomes depending on dose, exposure route, and physiological differences among species. Its widespread presence in industrial waste, soil, and water raises concerns about ecological health and food chain contamination. Figure 3 presents a comparative overview of vanadium toxicity in humans and animals, highlighting the key health effects across various species.

##### 4.3.2.1. Species-specific sensitivity

Animal susceptibility to vanadium varies significantly among species, reflecting differences in metabolic rate, gastrointestinal absorption, and detoxification mechanisms. Avian species, especially poultry, are among the most sensitive to dietary vanadium exposure. Toxicity has been reported at concentrations as low as 100 mg kg^−1^ in feed, with adverse effects increasing at levels approaching 800 mg kg^−1^ [73].

##### 4.3.2.2. Clinical manifestations

Exposure to high levels of vanadium causes obvious clinical damage in various organ systems. Gastrointestinal disturbances are frequently reported, including diarrhea, vomiting, and anorexia, which can lead to rapid weight loss and dehydration. In severe exposure situations, systemic toxicity advances to hematuria and hemorrhagic diarrhea. Notably, livestock grazing near vanadium-mining operations have shown these signs, leading to fatal outcomes [85]. These clinical symptoms are indicative of underlying mucosal damage, oxidative stress, and dysfunction of the renal and hepatic systems.

##### 4.3.2.3. Reproductive toxicity

The reproductive system also seems highly susceptible to damage caused by vanadium in animal studies. Male reproductive health has been well documented, with histopathological evaluations showing degeneration of seminiferous tubules and interference with spermatogenesis. Such damage is linked to decreased sperm viability and lower fertility. The observed effects are caused by vanadium’s disruption of hormonal balance, oxidative damage to reproductive tissues, and direct toxicity to germ cells. Although female reproductive outcomes are less studied, early findings indicate possible risks to embryonic development and pregnancy support [86].

The analytical difficulty of precisely quantifying individual species in complex matrices is shown by the substantial dependency of vanadium toxicity on oxidation state and chemical form. Because biologically significant speciation is frequently obscured by conventional total metal assays, selective extraction, hyphenated procedures, and oxidation state-sensitive detection approaches are required.

## 5. Vanadium speciation and determination

**Figure 4:**
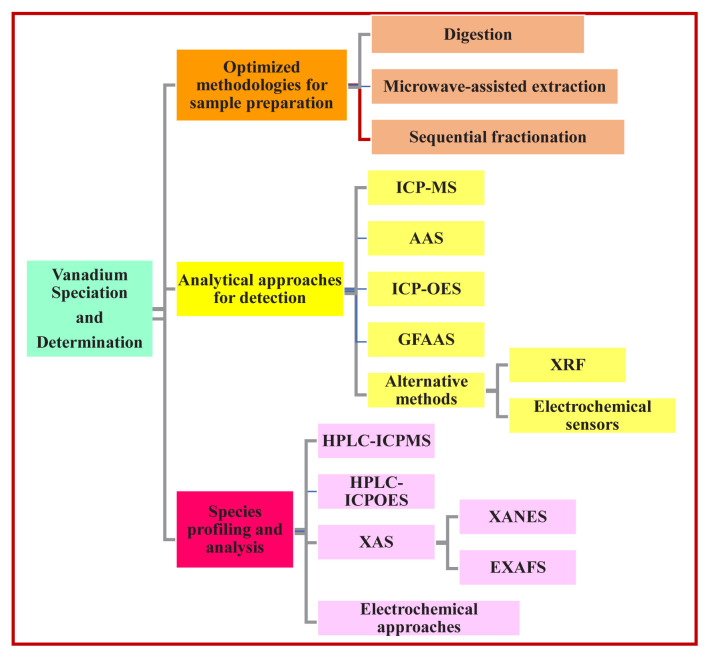
Conceptual flow chart mapping vanadium speciation and selective analytical determination pathways.

Accurate quantification and speciation of vanadium in environmental samples are essential for strengthening our understanding of its biogeochemical cycling, bioavailability, and associated toxic effects. Several advanced analytical techniques are used for metal speciation and detection [87]. Due to vanadium’s complex redox behavior and its typically trace-level concentrations in natural matrices, analytical methods must possess exceptional sensitivity, selectivity, and reliability to ensure precise detection and characterization [62]. Figure 4 illustrates the speciation of vanadium and the analytical methods used for its detection and quantification.

### 5.1. Optimized methodologies for sample preparation

Environmental matrices, including water, soil, sediment, and biological samples, require thorough pretreatment procedures to effectively extract vanadium while preserving its original speciation. Sample preparation is essential for analytical methods, especially for vanadium species, which are difficult to examine because of their low quantities and complicated matrices in biological and environmental materials. Solvent extraction and solid-phase extraction are the two primary methods for removing matrix components, which is frequently required. For vanadium speciation analysis, solvent-phase extraction is recommended over conventional solvent extraction in contemporary laboratories, whether employing offline or online preconcentration techniques [62].

The technique of liquid–liquid extraction (LLE), which depends on the formation of uncharged species in an aqueous phase, entails moving an analyte between two immiscible solvents. To improve selectivity for the target analyte while reducing other factors, the distribution ratio that defines the analyte’s equilibrium concentration in organic versus aqueous phases is essential. Effective extraction techniques, such as the selective removal of matrix materials or the optimization of solvent selection based on pH and miscibility, boost recovery and enrichment factors. Particularly for vanadium species, LLE is remarkably successful in preparing samples for atomic spectroscopy detection. Acid digestion, microwave-assisted extraction, and sequential fractionation are some of the other established methods commonly used to classify vanadium into operationally defined chemical forms [88–92].

### 5.2. Analytical approaches for detection

For determining total vanadium at trace levels, inductively coupled plasma mass spectrometry (ICP-MS) remains the definitive analytical method due to its exceptional sensitivity, wide dynamic range, and ability for simultaneous multielement analysis. Polyatomic and isobaric interferences have been effectively minimized through recent advancements, specifically the integration of collision or reaction cell technology, which has improved analytical accuracy and detection limits. Moreover, when paired with chromatographic techniques, ICP-MS enables speciation analysis, offering valuable insights into the chemical forms of vanadium in complex environmental systems [93–95]. Furthermore, both inductively coupled plasma optical emission spectroscopy (ICP-OES) and atomic absorption spectroscopy (AAS) are commonly used for measuring total vanadium in biological and environmental matrices because of their ease of use, sensitivity, and selectivity [62,96].

Vanadium can be detected at trace quantities using graphite furnace atomic absorption spectrometry (GFAAS), which frequently achieves sensitivity below the ppb level. This makes GFAAS an excellent tool for examining vanadium in biological tissue, soil, and water samples. According to Nowka et al. [97] and Garcia et al. [98], however, single-element analysis and very low sample throughput limit its usefulness. In contrast, ICP-OES provides stable performance, higher analytical throughput, and simultaneous multielement detection, with detection limits usually in the low ppb range. According to Hu and Coetzee [96], this makes ICP-OES a very suitable tool for large-scale environmental monitoring projects. To ensure complete conversion of vanadium species into detectable forms, both methods are typically used after sample digestion, either through acid treatment or microwave-assisted procedures. However, a major limitation of these methods is their inability to distinguish between vanadium species, which is crucial for evaluating the metal’s environmental behavior, movement, and toxicity. Alternative techniques such as X-ray fluorescence [99,100] and electrochemical sensors [101] have also been investigated for vanadium detection, although these methods generally offer lower sensitivity or require extensive sample preparation.

Spectrophotometric methods offer simplicity and low-cost instrumentation, facilitating simultaneous determination of V(IV) and V(V) with separation techniques. However, their detection limits of 0.1 to 0.5 mg L^−1^ are inadequate for environmental samples, making direct spectrophotometric determination unsuitable for such applications without prior sample treatment. Because vanadium fluxes in environmental samples are low, techniques like ICP or ICP-OES combined with ion exchange chromatography (IC) are preferred as they improve detection limits with element information that is specific, sensitive, and selective. ICP-MS is preferred for identifying vanadium species due to its multiisotope detection and higher sensitivity compared to ICP-OES, despite disadvantages including equipment cost and polyatomic ion interference.

Covalently bonded element species with distinct valence states, such as V(IV) and V(V) or Cr(III) and Cr (VI), are successfully separated using IC. Vanadium can be separated quickly and effectively using capillary electrophoresis (CE), which has advantages over IC, including reduced costs and higher separation efficiencies. However, CE has trouble with lower detection limits utilizing UV detection, which makes it less appropriate for determining trace amounts of vanadium in environmental materials. As a result, it must be coupled with more sensitive detection methods like ICP-MS or preconcentration techniques.

### 5.3. Speciation profiling and analysis

Vanadium speciation is commonly analyzed using hyphenated techniques that combine chromatographic separation with element-specific detection. Since pH and redox potential affect vanadium speciation, sample storage is essential for correct vanadium speciation in high-performance liquid chromatography (HPLC)-ICP-MS measurements. Incorrect handling can change V(IV) to V(V), producing speciation profiles that are deceptive. Vanadium speciation can be maintained for 6–7 days with minor concentration variations (<10%) by immediate filtration and chilled storage at 4 °C with the native pH. In contrast, common chemical preservation methods like acidification may distort speciation by causing redox transformations or complexation. For accurate and comparable speciation data, low-temperature storage, quick analysis, and meticulous reporting of preservation procedures are essential [102].

HPLC coupled with ICP-MS or ICP-OES helps to distinguish vanadium species based on charge, size, or complexation state, allowing effective differentiation between V(IV) and V(V) oxidation states in water-based environments [103,104].

X-ray absorption spectroscopy (XAS), encompassing both X-ray absorption near edge structure (XANES) and extended X-ray absorption fine structure (EXAFS) techniques, offers direct in situ insights into the oxidation state and local coordination geometry of vanadium within both solid and liquid matrices [87,105]. Electrochemical approaches such as anodic stripping voltammetry provide highly sensitive speciation analyses but demand rigorous control over experimental parameters to ensure data reliability [106].

### 5.4. Innovative approaches in vanadium analysis

Recent developments involve the use of synchrotron-based techniques that enable in situ analysis at environmentally relevant concentrations and spatial scales, as well as the coupling of capillary electrophoresis with ICP-MS, which enhances resolution for vanadium speciation in complex samples [106,107]. At the same time, advancements in portable sensor technologies and microextraction methods [108,109] are enabling the rapid on-site monitoring of vanadium in various environmental settings [77,110,111]. Table 2 summarizes the primary analytical methods used for identifying and quantifying vanadium in multiple matrices.

## 6. Regulatory standards and environmental guidelines

Despite the importance of vanadium in environmental and toxicological contexts, regulatory frameworks remain inadequate and inconsistently enforced. Table 3 lists vanadium exposure limits set by major U.S. agencies, including ceiling values and minimal risk levels (MRLs) for different exposure durations. Variations in allowable exposure limits, the lack of standardized protocols for speciation, and gaps in comprehensive risk assessment strategies have all contributed to its classification as an emerging yet insufficiently regulated contaminant.

### 6.1. Permissible limits of vanadium in potable water

Vanadium is not subject to consistent regulatory limits in drinking water across different jurisdictions. The World Health Organization has refrained from establishing a formal guideline. However, individual countries have established national standards or proposed health-based targets. In the United States, the Environmental Protection Agency (EPA) has listed vanadium on its Contaminant Candidate List, but no federal maximum contaminant level has been established [121]. Notably, California has established a public health goal of 50 μg/L, whereas Health Canada has proposed a guideline value of 60 μg/L for vanadium in drinking water [122,123].

### 6.2. Environmental benchmarks for vanadium in soil and sediments

Vanadium soil concentration thresholds vary significantly across different jurisdictions and often depend on land-use classifications. For example, the Netherlands has set target and intervention values of 42 mg kg^−1^ and 210 mg kg^−1^, respectively, while China’s environmental quality standard for agricultural soils limits vanadium at 100 mg kg^−1^ [123,124]. These regulatory standards reflect the total vanadium content but do not account for its speciation or biological availability.

### 6.3. Occupational threshold values for vanadium exposure

The acceptable exposure levels of vanadium set by regulatory and advisory bodies vary significantly, reflecting differences in assessments of the metal’s potential risks to occupational health. Organizations such as OSHA, NIOSH, ACGIH, ATSDR, and the EU SCOEL have established time-weighted averages or ceiling limits based on specific factors, including respiratory toxicity, chemical form, and exposure duration. As a very lax occupational standard, OSHA sets the maximum permissible ceiling concentration for vanadium pentoxide fume at 0.1 mg m^−3^. On the other hand, to protect against acute inhalation effects, NIOSH, ACGIH, and EU SCOEL recommend stricter limits of 0.05 mg m^−3^. ATSDR sets the most stringent benchmark with a chronic MRL of 0.0009 mg m^−3^, informed by prolonged exposure data from animal studies.

**Figure 5:**
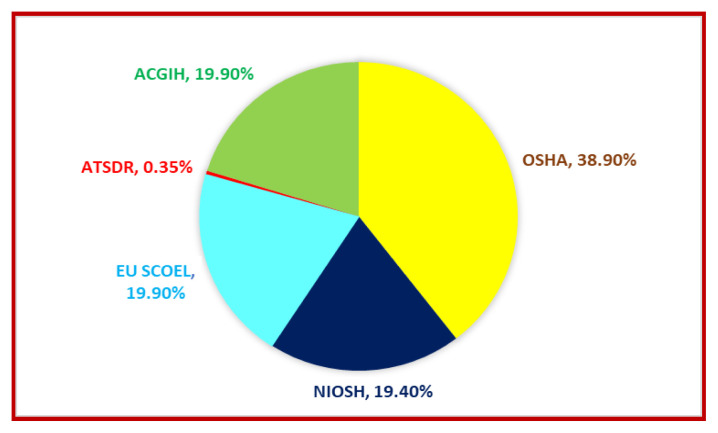
Occupational exposure limits for vanadium across regulatory and guideline frameworks. See Table 3 for acronyms.

Comparative analysis reveals that OSHA’s limit accounts for approximately 39% of the combined regulatory threshold values, whereas ATSDR’s share is less than 1%, highlighting the distinct differences between occupational safety approaches and long-term public health risk assessments [125]. Figure 5 illustrates the distribution of occupational limits for vanadium exposure across various guidelines, expressed as percentages.

### 6.4. Outstanding challenges and emerging opportunities

Significant challenges remain due to the lack of standardized environmental threshold values for vanadium, the limited incorporation of speciation data into risk assessments, and reliance on outdated toxicity reference values. Many existing guidelines are based on legacy data that do not reflect recent advances in understanding vanadium’s redox-dependent toxicity and bioavailability. Furthermore, current regulatory frameworks often fail to recognize emerging vanadium sources, such as redox flow batteries, electronic waste, and agricultural runoff.

Future regulatory efforts should prioritize the following:

1. The incorporation of vanadium speciation into environmental monitoring and risk evaluation protocols;
2. The development of harmonized quality standards for vanadium across air, water, and soil compartments; and
3. Enhanced collaboration between scientific communities and policymakers to update and refine existing guidelines.

## 7. Representative case studies of vanadium contamination

Case studies from various geographic areas illustrate the environmental persistence, spatial distribution, and ecological impacts of vanadium contamination, especially near industrial sites and mining operations. A summary of reported vanadium contamination is provided in Table 4.

### 7.1. Vanadium contamination in mining regions

Elevated vanadium levels have been observed in soil and water around the Bushveld Complex in South Africa, a major global vanadium reserve. Reported concentrations in surface waters and sediments surpass natural background levels by several orders of magnitude, with bioaccumulation seen in local aquatic organisms [126,127]. These findings highlight potential long-term ecological effects and risks to human health through water consumption and trophic transfer.

### 7.2. Industrial emissions and urban pollution

Studies conducted in metropolitan areas such as Shanghai, China, and Houston, USA, link airborne vanadium particle pollution mainly to fossil fuel burning and metallurgical activities. Vanadium levels in fine particulate matter (PM_2.5_) are significantly higher, creating respiratory health risks for exposed populations. Later deposition onto soils and water systems helps spread vanadium contamination across different environmental areas [128,129].

### 7.3. Environmental implications of vanadium redox flow batteries

Preliminary investigations from pilot-scale VRFB installations in Europe suggest possible environmental releases of vanadium via accidental electrolyte leakage and disposal practices [130,131]. Although current data are limited, these findings highlight the necessity for robust environmental monitoring and risk mitigation strategies concurrent with VRFB deployment. New routes for environmental release and redistribution are created by the growing industrial use of vanadium, especially in the manufacturing of steel and VRFBs. The necessity of assessing industrial life cycles in addition to environmental outcomes and transport mechanisms is highlighted by the potential for these human inputs to change local speciation, mobility, and bioavailability.

VRFBs pose unique environmental and toxicological hurdles for the disposal and end-of-life management of their electrolytes. These challenges go beyond operational considerations and encompass broader issues of chemical stability and vanadium speciation. Concerns are being raised regarding the stability of VRFB electrolytes, which are made of concentrated vanadium solutions in strong acids, as well as possible risks if they are disposed of improperly. Life-cycle assessments show that waste management and production contribute significantly to acidification and ecotoxicity, although disposal routes are still unclear [132–134]. Recycling is feasible; however, problems like impurity buildup, electrolyte aging, and redox imbalance make regeneration difficult and produce secondary waste. Because vanadium species are prone to precipitation and transformation under different conditions, which may result in enhanced mobility and toxicity, the chemical stability of these electrolytes can create environmental risks [135,136]. VRFB electrolyte disposal and leakage pose resource-management and toxicological challenges, necessitating life-cycle assessments, containment strategies, and regulatory frameworks for vanadium species in environmental contexts.

## 8. Prospects and emerging research frontiers

As vanadium shifts from being a trace element to an acknowledged environmental contaminant, it is crucial to address key research gaps to improve risk assessment and enable sustainable management.

### 8.1. Microbial contributions to biogeochemical interactions

Microorganisms play a crucial role in the redox cycling of vanadium, significantly affecting its speciation, mobility, and bioavailability. However, there is a lack of knowledge about microbial-based vanadium redox studies, and new cutting-edge molecular and isotopic methods are necessary [43]. Additionally, an exciting area for future research is exploring the potential microbial mechanisms for vanadium restoration.

### 8.2. Persistent toxicological and ecological threat evaluation

Integrative research combining molecular-level spatial analysis with ecosystem-scale evaluations is necessary to advance our understanding of vanadium’s environmental behavior. Prospective research should focus on developing innovative remediation technologies, evaluating long-term ecological impacts, and clarifying the microbial mechanisms responsible for vanadium redox conversions (Table 5). Emphasizing advanced analytical techniques alongside interdisciplinary approaches will be essential to fill knowledge gaps and minimize vanadium contamination [20].

### 8.3. Environmental outcomes of circular economy strategies

Vanadium can be recovered and reprocessed from discarded batteries and industrial effluents; however, there are significant environmental and technical challenges to overcome. A thorough assessment of these processes’ impact on the environment is crucial, especially considering the risk of secondary contamination and interference with the natural biogeochemical cycle of vanadium. A systematic approach for enhancing resource security and sustainability is to recover vanadium from such secondary sources. This method finds applications in secondary metallurgical operations and VRFBs. This approach also reduces dependence on primary ore extraction and its associated environmental impacts. Recycling vanadium may lessen ecotoxicity and life-cycle toxicity. Depending on system boundaries and assumptions, reductions of up to ~50% have been recorded in specific circumstances, such as the creation of battery-grade electrolytes. The outcomes of such studies indicate that the reprocessing of vanadium can lead to a reduction in ecotoxicity levels, in line with life-cycle toxicity [142–144]. Vanadium recycling can have negative environmental impacts, however, despite its potential benefits, especially when methods such as hydrometallurgical leaching or thermal processing are employed. Land and aquatic habitats may be negatively impacted by these methods, including effects such as the production of acidic effluents, the discharge of heavy metals, and the accumulation of process residues [145–147]. Anthropogenic extraction and recycling of vanadium could also disrupt its normal biogeochemical cycling. These challenges highlight the need for careful monitoring and management, since anthropogenic vanadium fluxes can surpass natural background contributions by about 70% in highly industrialized areas, according to a number of studies; estimates are highly dependent on methodological assumptions and geographical activity [9,10]. The existing research on vanadium recycling studies is often limited by the lack of real-world data on emissions and effluent profiles, with several life cycles relying on theoretical assumptions [148–150]. There remains a critical need to evaluate long-term ecological risks, secondary contamination, and effects on vanadium speciation and mobility within environmental matrices.

### 8.4. Harmonization of analytical methods and regulatory frameworks

The harmonization of analytical methods and regulatory frameworks is essential to ensure consistency and comparability in vanadium monitoring and risk assessment across different regions and studies.

The Organisation for Economic Co-operation and Development’s Good Laboratory Practice (OECD GLP) framework requires consistent procedures for toxicity studies and encourages international data acceptance. Studies complying with GLP and OECD testing guidelines in one member country are recognized by others, thereby minimizing duplication [151,152].

Harmonization ensures that analytical outcomes are comparable across laboratories by identifying and adjusting for intermethod discrepancies. Strategies such as round-robin testing, external proficiency schemes, and the use of certified reference materials with SI-traceable calibration standards are crucial for ensuring data traceability and consistency in analysis [153,154].

EPA-endorsed analytical procedures, formulated through public participation and regulatory review, are established as standardized procedures that consistently, precisely, and reproducibly demonstrate their effectiveness across environmental compliance initiatives [116].

## 9. Conclusion

Vanadium is increasingly being recognized as a global pollutant that was previously overlooked as a trace element. The main reason for this shift is its expanding industrial use, especially in the metallurgical and energy storage sectors, which have raised human emissions and disturbed its normal geochemical cycle. Effective environmental monitoring, toxicity assessment, and regulatory control are all complicated by these changes. This study has highlighted the intricate environmental behavior of vanadium, which greatly impacts ecosystems and human health. These behaviors are shaped by the metal’s diverse chemical forms, active biogeochemical cycle, and inputs from both natural and anthropogenic sources. Although recent advances in analytical techniques have improved the accuracy of vanadium detection and speciation, standardized methods and comprehensive regulatory frameworks are still lacking.

Future research must emphasize filling vital information gaps, especially in the areas of chronic toxicological effects, microbial-mediated transformations, and the environmental implications of recycling technology. The integration of detailed speciation risk evaluations with harmonized regulatory frameworks provides a proactive approach for managing vanadium and addressing its ecological and public health hazards. When taken as a whole, vanadium speciation functions as a unifying phenomenon that connects environmental occurrence, analytical determination, toxicological behavior, and industrial use. Accurately evaluating vanadium’s environmental risk in light of rising anthropogenic demand requires including these aspects of its use.

## Figures and Tables

**Table 1:** Representative vanadium-containing minerals.

Mineral name	Chemical formula	Remarks
Vanadinite	Pb_5_(VO_4_)_3_Cl	Lead chlorovanadate, found in oxidized Pb deposits
Patrónite	VS_4_	Sulfide ore, main vanadium ore in Peru (Minas Ragra)
Volborthite	Cu_3_V_2_O_7_(OH)_2_·2H_2_O	Oxidized Cu-vanadium mineral
Vanoxite	V^4+^_4_V^5+^_2_O_13_·8H_2_O	Rare vanadium oxide mineral
Fianelite	Mn_2_V(V,As)O_7_·2H_2_O	Manganese vanadate, found in metamorphosed ores
Vanalite	NaAl_8_V_10_O_38_·30H_2_O	Rare hydrated sodium-aluminum vanadate
Roscoelite	K(V^3+^,Al,Mg)_2_(AlSi_3_O_10_)(OH)_2_	Vanadium mica, common in black shales and sandstones
Carnotite	K_2_(UO_2_)_2_(VO_4_)_2_·3H_2_O	Uranium-vanadium ore, common in sandstones
Titanomagnetite	(Fe^2+^,Fe^3+^)(Fe^3+^,Ti)_2_O_4_	Igneous origin in layered mafic intrusions such as Bushveld Complex, South Africa

**Table 2:** Analytical methods for vanadium detection and speciation, outlining limit of detection (LOD), compatible matrices, speciation performance, and principal strengths and constraints.

Technique	LOD	Speciation	Matrix	Advantages	Limitations
ICP-MS [62]	~ng L^−1^	No	Water, soil, biota	High sensitivity, multielemental	Needs digestion, costly
ICP-OES [112]	~μg L^−1^	No	Water, soil, sediments	Good for total V, less expensive, multielemental	Limited speciation, lower sensitivity than ICP-MS
AAS (GFAAS) [113]	~μg L^−1^	No	Water, digested solids	Widely available, simple	Single-element, moderate sensitivity
XAS/XANES [105]	~ppm	Yes	Solids, sediments	In situ speciation	Requires synchrotron access
NAA [113]	~μg L^−1^	No	Biological and environmental samples	Very sensitive, multielemental	Time-insensitive
LC-ICP-SFMS [113]	Low, ~ng L^−1^	Yes	Water (environmental)	High sensitivity	Requires chromatographic separation
Spectrophotometric or kinetic methods [114]	~0.3 μg L^−1^	No	Water	Simple and inexpensive	Narrow linear range
Electrochemical (ASV, CSV, voltammetry) [115]	Low, ~μg L^−1^	Yes	Aqueous environmental samples	Sensitive, field-portable, speciation	Needs electrode optimization, limited scope

**Table 3:** Summary of vanadium exposure limits from major U.S. agencies, expressed in mg m^−3^ as ceiling values or minimal risk levels (MRLs) across different exposure durations citing insufficient toxicological evidence.

Agency or guideline	Exposure limit (mg m^−3^)	Exposure type	Remarks
Occupational Safety and Health Administration (OSHA) [116]	0.5	Ceiling limit (8-hour time-weighted average)	Vanadium pentoxide (V_2_O_5_)
National Institute for Occupational Safety and Health (NIOSH) [117]	0.05	Ceiling limit (15-min time-weighted average)	Vanadium compounds, except metal and carbide
American Conference of Governmental Industrial Hygienists (ACGIH) [118]	0.05 (fume), 0.1 (dust)	Threshold limit value - ceiling	Vanadium pentoxide (V_2_O_5_), based on irritation and lung damage risk
EU Scientific Committee on Occupational Exposure Limits (EU SCOEL) [119]	0.05	Time-weighted average	Proposed based on chronic inhalation toxicity
Agency for Toxic Substances and Disease Registry (ATSDR) [120]	0.0008	Acute-duration inhalation MRL	Based on respiratory effects in animal studies
	0.0001	Chronic-duration inhalation MRL	

**Table 4:** Global distribution of reported vanadium contamination by source and impacted environmental media.

Source	Location	Environmental media	Key findings
Vanadium mining	Bushveld Complex, South Africa	Soil, surface water, sediments, aquatic biota	Vanadium fluxes several-fold above background; bioaccumulation in aquatic organisms; ecological and human exposure concerns
Industrial emissions	Shanghai, China; Houston, USA	Air (PM_2.5_), soil, water	Elevated airborne vanadium particulate from fossil fuel combustion and metallurgical sources; respiratory health risks; soil and water contamination
Vanadium redox flow battery facilities	Europe	Soil, water (electrolyte leakage)	Potential vanadium release through leakage and disposal; limited data; calls for environmental monitoring

**Table 5:** Toxicological effects of vanadium on different organisms [42,137–141].

Organism	Endpoint	Effect level (LC_50_/EC_50_/NOEC)	Speciation tested
*Daphnia magna*	Immobilization	0.2 mg L^−1^ (LC_50_, 48 h)	V(V)
*Raphidocelis subcapitata*	Growth inhibition	0.05 mg L^−1^ (EC_50_, 72 h)	V(V)
*Eisenia fetida*	Reproduction rate	NOEC: 70 mg kg^−1^	V(IV)
*Arabidopsis thaliana*	Root length	Inhibited at >5 mg L^−1^	V(V)
Human (*in vitro*)	DNA damage/ROS generation	Observed at >0.01 mM	V(IV), V(V)
